# Identification of drought stress related proteins from 1S^l^(1B) chromosome substitution line of wheat variety Chinese Spring

**DOI:** 10.1186/s40529-016-0134-x

**Published:** 2016-08-09

**Authors:** Jiaxing Zhou, Chaoying Ma, Shoumin Zhen, Min Cao, Friedich J. Zeller, Sai L. K. Hsam, Yueming Yan

**Affiliations:** 1 College of Life Science, Capital Normal University, Beijing, 100048 People’s Republic of China; 2 Division of Plant Breeding and Applied Genetics, Technical University of Munich, 85354 Freising-Weihenstephan, Germany

**Keywords:** 2-DE, Proteome, Wheat, Drought tolerance, *Aegilops longissima* 1S^l^ chromosome

## Abstract

**Background:**

Wheat, one of the most important crops, has a detrimental effect on both yield and quality under drought stress. As our preliminary experiment showed that the Chinese Spring wheat-*Aegilops longissima* chromosome substitution line CS-1S^l^ (1B) had a better drought tolerance than CS, the substitution line CS-1S^l^(1B) was used to identify drought stress related proteins by means of a comparative proteome approach in this work. Our present study aimed to explore the gene resources for drought resistance in 1S^l^ genome.

**Result:**

Our results showed that drought stress induced downregulation of relative water and chlorophyll contents and the upregulation of proline content, and further influencing grain filling shortening and significant decrease of plant height, B-type starch granule numbers, grain number and weight. In total, 25 grain albumin and globulin protein spots were found to be specifically encoded by the 1S^l^ chromosome. In addition, 17 protein spots respected 13 unique proteins were identified by MALDI-TOF/TOF MS, which were mainly involved in adverse defense and gluten quality. Among them, ascorbate peroxidase, serpin-Z2B and alpha-amylase/trypsin inhibitor were upregulated under drought stress. These proteins play important roles in plant drought defenses through various metabolic pathways.

**Conclusion:**

Our results indicate that the 1S^l^ chromosome of *Aegilops longissima* has potential gene resources that could be useful for improving wheat drought resistance.

**Electronic supplementary material:**

The online version of this article (doi:10.1186/s40529-016-0134-x) contains supplementary material, which is available to authorized users.

## Background

Drought is well known for its detrimental effects as a major consequence of extreme climate, causing significant decrease in both yield and quality in landraces and wild relatives of crop species during grain filling (Boyer et al. 2004; Feuillet et al. 2008; Dodig et al. 2012). As one of the most important crops and the main food source for the world population, wheat can have a complex and powerful reflect facing drought stress. To improve the resistance of wheat to drought and minimize the damage, it is highly important to understand the mechanism of drought stress process and explore new gene resources for the improvement of drought resistance.

In the condition of drought stress, the various stages of plant growth and development would be impacted. Water stress during the grain-filling period usually induces early senescence and shortens the grain-filling period, due to the acceleration of carbohydrate reserving from the vegetative tissues to the grain (Yang et al. 2006). Drought stress is an osmotic effect, many mechanisms were involved in enhancing the drought resistance in plants. The proteins closely related to oxidation, stress and defense play critical roles in this process such as ascorbate peroxidase (APX). APX can reduce the accumulation of reactive oxygen species (ROS). The upregulated expression of APX can be seen as an antioxidative defense in plants.

Along with greater advance for wheat genomics (Brenchley et al. 2012; Ling et al. 2013; Mayer et al. 2014), considerable work from different omics levels of wheat had been reported recently. A fine transcriptome map of the chromosome 3B was constructed, and the new insights into the relationships between gene and genome structure and function were presented (Pingault et al. 2015). In recent years, different proteomic analysis for wheat roots, stems, leaves, and developing grains under the condition of water depletion have been investigated (Bazargani et al. 2011; Ford et al. 2011; Ge et al. 2012; Hao et al. 2015). These studies provided an important theoretical basis for understanding the drought stress response mechanism of wheat.

By means of distant hybridization and chromosome engineering, valuable genes from *Aegilops* and other related wheat species can be introgressed into wheat genome to enrich the germplasm resources and enhance the adversity resistant ability. *Aegilops* species has attracted much attention since it has desirable gene resources and is widely used for wheat drought-resistance improvement (Zaharieva et al. 2001; Molnár et al. 2004). Particularly, *Aegilops longissima* (2n = 2x = 14, S^l^S^l^) was shown to have eyespot and pre-harvest sprouting resistance (Sheng et al. 2012; Singh et al. 2013), and superior glutenin genes (Wang et al. 2013). However, the gene resources for drought resistance in 1S^l^ genome is not yet being explored and utilized so far.

In the present study, we investigated the specifically encoded proteins of the 1S^l^ chromosome in seeds and their responses to drought stress by using a comparative proteomics approach. Some key grain albumins and globulins involved in drought stress were identified. Our results demonstrated that the 1S^l^ chromosome has potential gene resources resistant to drought stress, which might be valuable for wheat improvement of drought resistance.

## Methods

### Plant materials, planting and drought treatment

The Chinese spring (CS) substitution line CS-1S^l^(1B) developed in Institute for Plant Breeding, Technical University of Munich, Germany was used as material, in which the 1S^l^ chromosome from *Aegilops longissima* (2n = 2x = 14, S^l^S^l^) was substituted for 1B of CS. The development procedures of CS–1S^l^(1B) were detailedly described in our previous work (Wang et al. 2013). In brief, CS was crossed with *Ae. longissima*, the F1 plants were treated by colchicine and CS-*Ae. longissima* amphiploid was obtained. Afterwards, an addition line (wheat + 1S^l^ chromosome pair) was appeared after the amphiploid backcrossed with CS for several times. The addition line was crossed with CS monosomic line (CS mono 1B) and the offspring was obtained. After self-pollination, the substitution line was developed.

Wheat seeds were put into 30 % sodium hypochlorite liquid for 20 min, then soaked overnight in 1 % hydrogen peroxide solution. The treated seeds were grown in the glasshouse at the Chinese Academy of Agricultural Sciences (CAAS), Beijing, from October, 2014 to January, 2015. Drought stress treatments during grain development included control and treated groups from tillering to mature stages, and each plot consisting of 200 plants. As the control group, we keep the soil moisture at 50 %, while the stress group at 20 %, approximately.

### Soil moisture measurement

To ensure the reliability of sustaining drought stress, soil water content from 20 cm was measured every ten days after sowing (DAS). Soil samples collected from three random spots of each replicate were put into aluminum boxes, and dried in an oven at 105 °C for 48 h. The soil moisture (W %) was calculated by the formula: W = (g_1_ − g_2_)/(g_2_ −g _0_) × 100 % (g_1_: the weight of the moist soil; g_2_: the weight of the dry soil; g_0_: the weight of the empty box).

### Measurement of leaf physiological parameters

Half a month after tillering, the relative water content (RWC), chlorophyll content and proline content of leaves were measured nearly every two weeks (on 42, 55, 69, 81, 93 and 105 DAS, respectively) based on Zhang (2014). During any measurement, leaves samples were collected from three random spots of each replicate. And three replicates were performed.

### Endosperm ultrastructure observation by scanning electron microscope (SEM)

Mature grains from both treatment and control groups were put in the fixative (5 ml 38 % formalin, 5 ml glacial acetic acid, 90 ml 70 % ethyl alcohol) for a minimum of 12 h. Then the grains were dehydrated sequentially in 70 % ethanol solutions (20 min), 80 % ethanol solutions (20 min), 90 % ethanol solutions (overnight) and 100 % ethanol solutions (20 min). The samples were treated stepwise for 20 min in mixtures of ethanol and isoamyl acetate with ratios 3:1, 1:1 and 1:3 before soaking in isoamylacetate. Finally, critical point drying was done for SEM observation. Grain endosperm ultrastructures were observed by scanning electron microscope S-4800 FESEM (Hitachi, Japan).

### Protein extraction, 2-DE and images analysis

Albumin and globulin proteins from mature grains were extracted according to Ge (2012). After extracting in lysis buffer (7 M urea, 2 M thiourea, and 4 % CHAPS), the concentrations of proteins were measured by 2-D Quant Kit (Amersham Bioscience, USA).

The extracted proteins (600 µg) were loaded in 360 µl of buffer (7 M urea, 2 M thiourea, 2 % w/v CHAPS, and 0.2 % bromphenol blue) containing 65 mM DTT and 0.5 % immobilized pH gradient buffer (pH 3–10) (GE Healthcare). pH 3–10 IPG strips (18 cm, nonlinear, GE Healthcare) and Ettan IPGphor system were used for IEF.

The first dimension IEF was performed following the manufacturer’s instructions (30 V for 12 h, 300 V for 1 h, 500 V for 1 h, 1000 V for 1 h, 3000 V for 1 h, and then focusing at 8000 V until 80,000 Vh at 20 °C). After treated with equilibration buffer, SDS-PAGE was run on 12 % gels including 0.4 ml of 30 % (w/v) acrylamide/methylene bisacrylamide, 0.25 ml of 1.5 M pH 7.8 Tris–HCl, 0.33 ml of deionized water, 10 μl of 10 % (w/v) SDS, 10 μl of 10 % (w/v) ammonium persulfate, and 0.6 μl of TEMED according to Ge (2012). Three biological repetitions were done for error control.

After electrophoresis, proteins were visualized by colloidal Coomassie Brilliant blue (CBB) staining (R-250/G-250 = 4:1), and destained by destaining solution (distilled water with 10 % ethonal and 10 % acetic acid). The images were scanned by GS-800™ Calibrated Densitometer (BIO-RAD). Image analysis was performed with ImageMaster 2D Platinum Software Version 7.0 (Amersham Biosciences). Only those with biological reproducible protein spots were considered as the specifically encoded proteins by the 1S^l^ chromosome. The specifically encoded proteins were selected for further tandem MS analysis.

### Protein identification through tandem mass spectrometry

The selected spots were cut from 2-DE gels and decolored by bleaching solution (50 % 25 mM NH_4_HCO_3_ and 50 % acetonitrile) in EP tubes. After the protein spots colorless, the decoloring liquid was discard and 100 μl acetonitrile was add to the EP tubes. After samples turned white, dry treatment was performed for at least 30 min. The dry samples were digested with 7 μl diluted solvent (trypsin enzyme solution diluted with 25 mM NH_4_HCO_3_, the final concentration 15 ng/μl), and incubated at 37 °C for at least 16 h. Subsequently, the peptides were extracted with 5 % trifluoroacetic acid (TFA), 50 % acetonitrile and 45 % water at 37 °C for 1 h. Extracts were dried using a vacuum dryer. The dried peptide mixtures were completely dissolved in 2 μl solution containing 0.1 % TFA mixed with 1 μl TFA, 500 μl acetonitrile solution and 499 μl double distilled water.

Tryptic peptides were analyzed with a MALDI-TOF/TOF mass spectrometer 4800 Proteomics Analyzer (Applied Biosystems, Framingham, MA, USA). All the MS/MS spectra were searched in the NCBI non-redundant green plant database. The peptide mass tolerance was 100 ppm, the fragment mass tolerance were 0.2 Da, allowed one missed cleavage. Carbamidomethyl (Cys) and oxidation (Met) were specified as variable modifications. Only MASCOT scores more than 65 (*p* < 0. 05) were accepted.

## Results

### Dynamic changes of soil moisture under drought stress

**Fig. 1:**
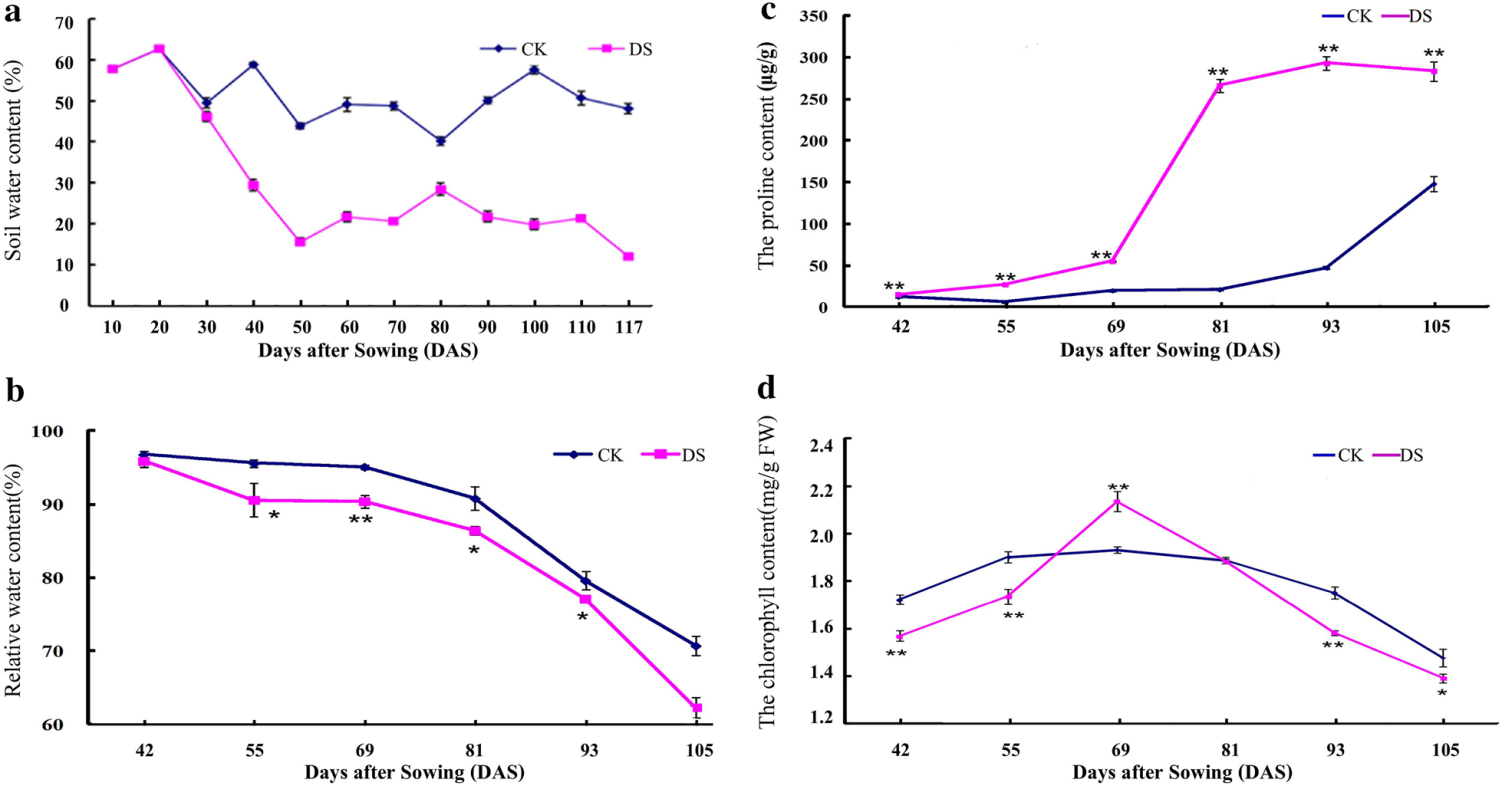
Soil water content changes and leaf physiological parameter changes. *CK* represents the control group, and *DS* represents the drought stress treated group. **a** Soil water content changes; **b** relative water content; **c** proline content; **d** chlorophyll content; * and**indicate a significant difference at *p* < 0.05 and *p* < 0.01 level by *t* test, respectively

The drought treatment effect was obvious after the tillering stage (28 DAS) of wheat. There was a great difference on the soil moisture between the control group and the treatment group (Fig. 1a). A sustaining severe drought stress was kept for the treatment group during whole grain developmental stages (soil moisture at approximately 20 %).

### Agronomic character, physiological parameter and grain ultrastructural changes under drought stress

Our preliminary experiment under drought stress showed that the substitution line CS-1S^l^(1B) had better drought tolerance than CS (Additional file 1: Figure S1). Compared to CS-1S^l^(1B), CS showed shorter grain filling time and ear length, smaller grain size and weight. This indicated that some drought related proteins from 1S^l^ chromosome were introgressed after 1B was substituted by 1S^l^ chromosome. Thus, in this study, we further performed a proteome analysis to identify the drought related proteins in CS-1S^l^(1B) introgressed from 1S^l^ chromosome.

Main agronomic trait changes of CS-1S^l^(1B) under normal cultivation and drought stress were shown in Additional file 2: Table S1 and Additional file 3: Figure S2. Drought stress resulted in shortening of grain filling time and significant decrease of main agronomic traits, including plant height, spike length, spikelet number, grain number and weight. These results indicate that drought reduces plant growth and dry matter accumulation through inhibiting photosynthesis (Yang et al. 2006; Hajheidari et al. 2007; Zhang et al. 2009).

Physiological parameter changes showed that relative water content (RWC) of leaves was down-regulated during grain development stages in both groups, but it was significantly lower in drought treated group (Fig. 1b). Contrary to RWC, proline content was remarkably up-regulated under drought stress (Fig. 1c), especially after 81 DAS. Proline plays an important role in plant defense as an osmotic agent. It is universally accepted that the content of proline in plant leaves could be increased under drought condition (Bowne et al. 2012; Zhang et al. 2014). In addition, drought stress let to a significant decrease of chlorophyll content except 69DAS with a reverse expression (Fig. 1d). The significant increase of chlorophyll content at this stage under drought condition is possibly due to the stress reaction, and the similar phenomenon was also observed previously (Izanloo et al. 2008).

**Fig. 2:**
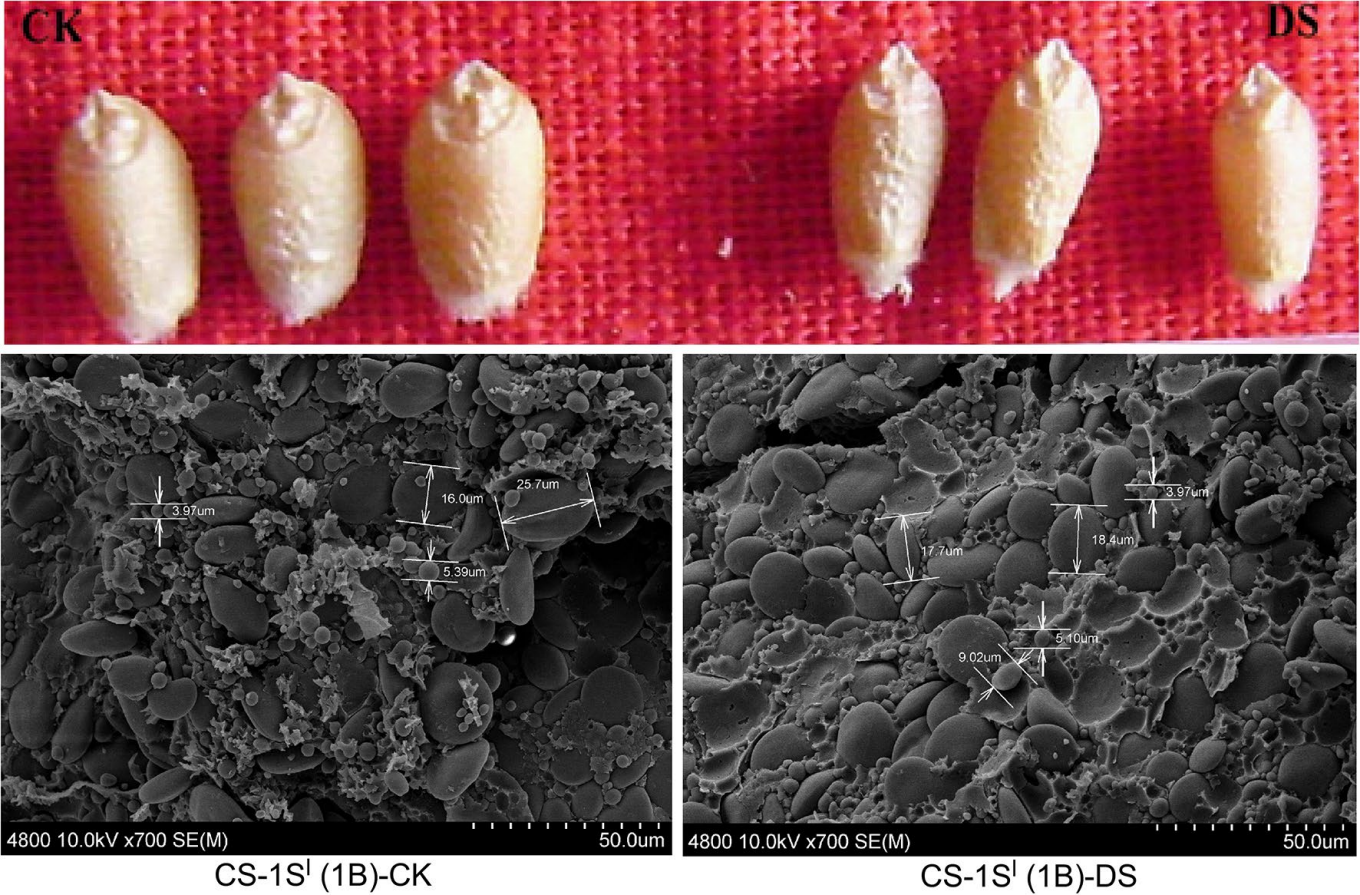
Grain morphology and SEM observation of CS-1S^l^ (1B) under well-watered and drought stress conditions. The scale is shown at the *bottom right corner* of the Figure, and one space is 5 μm, total is 50 μm

The ultrastructural characters of mature grain endosperm in both groups were observed by SEM (Fig. 2). Different types of starch granules could be clearly observed, including A-type starch granules with oval and more than 10 μm diameter and B-type starch granules with round and 5–10 μm diameter as well as a few smaller C-type starch granules with less than 5 μm diameter. Water stress reduces the formation of endosperm cells and starch granules, which limited the capacity of accumulating starch in endosperm (Nicolas et al. 1985; Saini and Westgate 2000). In line with this, less B-type starch granules were observed under drought stress, as the percent of B-type starch granules fell from 34.3 % to 15.1 %. While starch is the major storage carbohydrate in the seeds of cereal crops and comprises approximately 65–75 % of the weight of wheat grains (Hurkman et al. 2003). That may be a reason for the negative change of grain characters under drought stress, whether in size or weight (Fig. 2).

### Identification of 1S^l^-encoded proteins and their responses to drought stress

**Fig. 3:**
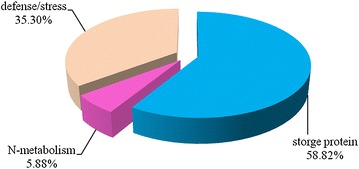
Functional distribution of 17 seed proteins encoded by 1S^l^ genome from CS-1S^l^ (1B)

According to 2-DE maps, 25 grain albumin and globulin protein spots (S1-S25) were found to be specifically encoded by the 1S^l^ chromosome through comparative proteome analysis between CS and CS-1S^l^(1B) (Additional file 4: Figure S3). Among them, 17 (68 %) protein spots including 13 unique proteins were successfully identified with a high degree of confidence by MALDI-TOF/TOF MS (Table 1, Additional file 5: Table S2), since there were some protein spots identified as the same protein. Three y-type high molecular weight glutenin subunit spots were found, two spots were identified as Globulin1 and Globulin2, respectively. Those proteins were grouped into three functional categories: defense/stress, N-metabolism and storage proteins (Fig. 3). Among them, five protein spots were identified as high molecular glutenin subunits (S2-S6) while the other five spots were identified as globulins (S7, S8, S9, S21, and S22).

**Table 1:** The proteins specifically encoded by 1S^l^ genome and their expression trends under drought stress

Spot ID	Accession no. (gi)	Protein description	Protein score	Protein score C.I. %	Total ion score	Total ion score C.I. %	Number of matching peptides	Sequence coverage %	TpI/MW (kDa)	EpI/MW (kDa)	Expression trend under drought stress
S2	39599016	HMW glutenin subunit	685	100	615	100	12	53.4	7.42/82.12	7.19/71.09	Downregulated
S3	140169817	Dy-type high molecular weight subunit protein	343	100	279	100	12	24.7	7.40/80.23	8.05/70.40	Downregulated
S4	344995121	Y-type high molecular weight glutenin subunit	381	100	331	100	11	20.7	7.35/79.32	7.28/80.63	Downregulated
S5	344995121	Y-type high molecular weight glutenin subunit	348	100	297	100	9	14.2	7.33/77.51	7.28/80.63	Downregulated
S6	344995121	Y-type high molecular weight glutenin subunit	390	100	339	100	11	20.6	7.17/78.21	7.28/80.63	Downregulated
S7	228310	Globulin 2	76	97.585	68	99.947	3	7.1	6.88/66.32	6.16/50.23	Downregulated
S8	34495244	Globulin-like protein	85	99.659	72	99.978	4	12.6	7.01/66.34	6.78/52.38	Downregulated
S9	228310	Globulin 2	84	99.608	76	99.992	3	7.1	6.82/60.10	6.16/50.23	Downregulated
S10	171027826	Triticin	563	100	487	100	13	30.7	6.75/60.22	6.43/65.29	Downregulated
S15	75279909	Serpin-Z2B	648	100	563	100	12	41.2	4.88/39.81	5.18/43.01	Upregulated
S17	584706	Aspartate aminotransferase	127	100	97	100	6	24.8	6.90/38.71	7.75/44.65	Downregulated
S20	259122791	APX	591	100	424	100	15	90.4	7.01/28.71	5.58/27.95	Upregulated
S21	110341790	Globulin 1	817	100	763	100	7	50.2	7.55/29.22	8.72/25.55	Upregulated
S22	110341790	Globulin 1	623	100	546	100	7	50.2	7.67/29.05	8.72/25.55	Upregulated
S23	21711	Alpha-amylase inhibitor CM 17 protein precursor	274	100	246	100	4	35.7	4.72/16.14	5.07/16.55	Upregulated
S24	221855632	Alpha-amylase inhibitor CM16 subunit	341	100	302	100	5	24.5	4.81/15.91	5.31/16.27	Upregulated
S25	123957	Alpha-amylase/trypsin inhibitor CM3	705	100	632	100	8	69.0	6.77/15.54	7.44/18.89	Upregulated

The protein spot S17 was identified as aspartate aminotransferase, which belong to metabolism related enzymes. The remaining 6 spots were identified as triticin (S10), serpin-Z2B (S15), APX (S20), alpha-amylase inhibitor CM 17 protein precursor (S23), alpha-amylase inhibitor CM16 subunit (S24) and alpha-amylase/trypsin inhibitor CM3 (S25). These proteins were mainly related to various biotic and abiotic stress defenses.

**Fig. 4:**
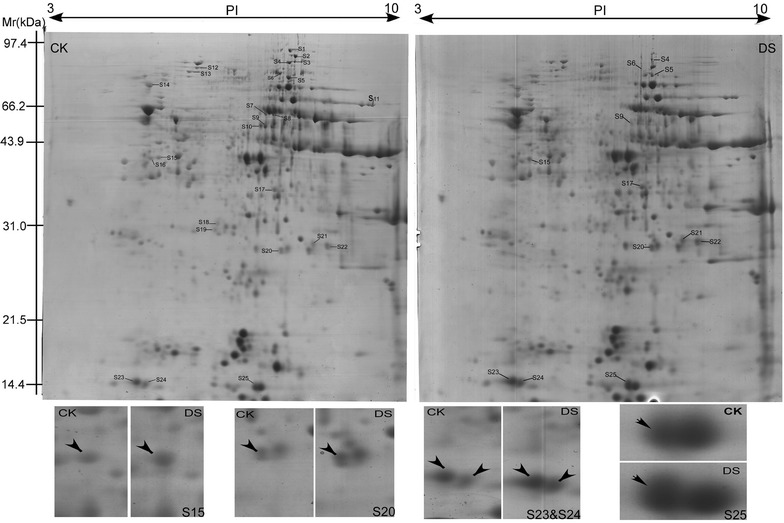
Proteome maps of CS-1S^1^ (1B) mature grains under well-watered (CK) and drought stress (DS) conditions. Sots S1 to S25 were the 1S^1^-encoded protein

Under drought stress, the expression of the 1S^l^-encoded proteins was detected (Fig. 4, Table 1). Several protein spots identified as glutenin subunits were downregulated (S2-6). The spot S7, S8, S9 which identified as globulin-2 or globulin-like protein were downregulated, while the spot S21 and S22 which identified as globulin-1 were upregulated. Triticin (S10) and aspartate aminotransferase (S17) were downregulated in this work. Some drought-related proteins showed upregulated expression, including APX (S20), serpin-Z2B (S15), alpha-amylase inhibitor CM 17 protein precursor (S23), alpha-amylase inhibitor CM 16 subunit (S24) and alpha-amylase/trypsin inhibitor CM3 (S25).

## Discussion

Drought stress research is always an important aspect for the resistance and quality study of wheat. To struggle with drought, many proteins in grains were involved in this stress resistance process. Among them, antioxidant enzymes were the common proteins. The contents of the common ROS-detoxifying enzymes, for instance peroxidase, superoxide dismutase and catalase, were generally upregulated under water deficit (Ge et al. 2012). In the previous work, the protease inhibitors such as alpha-amylase inhibitors and serpins were found induced by drought stress in grains (Jiang et al. 2012). As for the experimental material, CS substitution line CS-1S^l^(1B) is an achievement of chromosomal engineering, that showed to be a better breadmaking quality according to the previous work (Wang et al. 2013). However, the gene resources for drought resistance in 1S^l^ genome have not been explored. In this study, we investigated the specifically encoded proteins of the 1S^l^ chromosome and their responses to drought stress.

In terms of the functions of the identified proteins encoded by 1S^l^ chromosome, high molecular glutenin subunits (HMW-GS) were the important seed storage proteins imparting dough elasticity (Payne 1987), while globulins were not only the seed storage protein, but also the metabolism proteins with multiple functions. For instance, Altenbach suggest that both transcriptional and post-translational mechanisms are involved in the response of globulin-2 to high temperatures (Altenbach et al. 2009). As for the response to drought stress, our result demonstrated that the globulin-1 encoded by 1S^l^ chromosome showed an upregulated expression under the condition of water deficit, that can be a consequence of stress or an adaptation response under drought stress and might helpful for the stress resistance.

Several protein spots identified as glutenin subunits were downregulated, indicating that drought stress would decrease gluten content and breadmaking quality. APX was one of the drought-related proteins. ROS usually accumulates in plant cells under drought stress (Apel et al. 2004). APX works as a common ROS-detoxifying enzyme which can catalyze the conversion of H_2_O_2_ to H_2_O and O_2_, thus alleviate the acceleration of lipid peroxidation and leaf senescence caused by the high concentrations of H_2_O_2_ under drought stress (Upadhyaya et al. 2007). In line with this, APX showed an upregulated pattern under drought stress in this study.

Protease inhibitors generally express in storage tissues such as seeds after induction by adverse conditions (Koiwa et al. 1997; Van Dam et al. 2001; Dombrowski et al. 2003). They have a large and complex group and great diversity of functions in plants. Protease inhibitors can form a stable complex to regulate the activity of target protein (Leung et al. 2000), in which way to respond to a number of cellular physiological processes. Studies showed that some protease inhibitors induced by abiotic stress, and involved in the process of abiotic stress resistance in wheat (Shan et al. 2008) and other plants (Gaddour et al. 2001; Huang et al. 2007). Some of them involved in programmed cell death process regulation in plants, thereby improve the survival rate under the adverse conditions (Solomon et al. 1999). Thus, we speculate that the function of protease inhibitors in the abiotic stresses response is to inhibit the protease activity and maintain the stability of functional proteins and structural proteins in plant cells, then alleviate the secondary oxidation stress of abiotic stress on the toxicity of cells and improve the resistance of plants as previous reports (Orozco-Cárdenas et al. 2001; Shan et al. 2008).

Wheat serpins belong to the superfamily of serine protease inhibitors, they have been identified in almost all organisms (Silverman et al. 2001). Serpins usually have a reaction center loop (RCL), which protrudes out of its structure to recognize a particular target protease (Whisstock et al. 2007). Serpin family functions through irreversible inhibition of proteinases and play important roles in stress response (Roberts et al. 2008). In this work, the serpin-Z2B encoded by 1S^l^ chromosome showed an upregulated expression, therefore it was likely to play important roles in drought stress tolerance. Serpins as the defensive shield have the function of protecting the storage proteins from digestion (Vensel et al. 2005), which might be helpful to alleviate the decrease of storage proteins content in grains under drought stress. In line with this observation, previous research demonstrated that the downregulation of serpin gene exaggerated stress-induced cell death (Bhattacharjee et al. 2015). In addition, trypsin inhibitors were also common serine proteinase inhibitors. The role of jasmonic acid and abscisic acid treatments in alleviating drought stress and regulating trypsin inhibitor production in soybean was found, they proposed that the production of trypsin inhibitor in soybean plant could take place via a JA- or ABA-depending signaling pathway, as different concentrations of jasmonic acid and abscisic acid caused an accumulation of trypsin inhibitor in soybean leaves compared with the untreated control plants (Hassanein et al. 2009).

Our 2-DE results also showed that alpha-amylase inhibitors encoded by 1S^l^ genome showed an upregulated expression under drought stress in CS-1S^l^(1B). Alpha-amylase inhibitor was reported to play an important role in coping with biotic stress caused by insects (Franco et al. 2002). Furthermore, the alpha-amylase inhibitors can protect the starch reserves in the endosperm from degradation (Skylas et al. 2000) and improve the content and composition of gluten proteins during grain development under drought stress (Ge et al. 2012).

## Conclusion

This study found 25 grain albumin and globulin protein spots to be specifically encoded by the 1S^l^ chromosome. Among them, 17 protein spots representing 13 unique proteins were successful identified by MALDI-TOF/TOF MS. Our results from this study demonstrate that the 1S^l^ chromosome from *Aegilops longissima* has important proteins involved in adverse defense or gluten quality such as APX, serpin-Z2B, alpha-amylase inhibitor, trypsin inhibitor, HMW-GS and globulins. These proteins could be used as potential resources for improving wheat adverse resistance and breadmaking quality.

## Additional files

**Additional file 1: Figure S1.** Performance of drought tolerance between CS and CS-1S^l^ (1B).

**Additional file 2: Table S1.** Some agronomic character performance of CS-1S^l^ (1B) under drought stress and well-watered conditions.

**Additional file 3: Figure S2.** Pictures of CS-1S^l^ (1B) under drought stress and well-watered conditions in several grains development stages. (a).After tillering; (b). After harvest; (c). 5DPA; (d). 30 DPA.

**Additional file 4: Figure S3.** Proteome maps of wheat albumins and globulins from mature grains of CS and CS-1S^l^ (1B). S1 to S25 represented those specifically expressed in CS-1S^l^ (1B). The detail identification results were showed in Table1.

**Additional file 5: Table S2.** Peptide sequences of mature seed proteins encoded by 1S^l^ genome of CS-1S^l^(1B) identified by MALDI-TOF/TOF-MS.

## Abbreviations

CS: Chinese spring
SEM: scanning electron microscopy
2-DE: two-dimensional electrophoresis
IEF: isoelectric focusing
SDS-PAGE: sodium dodecyl sulfate–polyacrylamide gel electrophoresis
IPG: immobilized pH gradient
RWC: relative water content
MALDI-TOF/TOF-MS: matrix-assisted laser desorption/ionisation time-of-flight/time-of-flight mass spectrometry
DAS: days after sowing
HMW-GS: high molecular weight glutenin subunit
APX: ascorbate peroxidase
ROS: reactive oxygen species
JA: jasmonic acid
ABA: abscisic acid
