# Supplementary figures and images for: Identification of drought stress related proteins from 1Sl(1B) chromosome substitution line of wheat variety Chinese Spring
Source: Bot Stud. 2016 Aug 9;57:20. doi: 10.1186/s40529-016-0134-x (PMC5430570; doi:10.1186/s40529-016-0134-x)

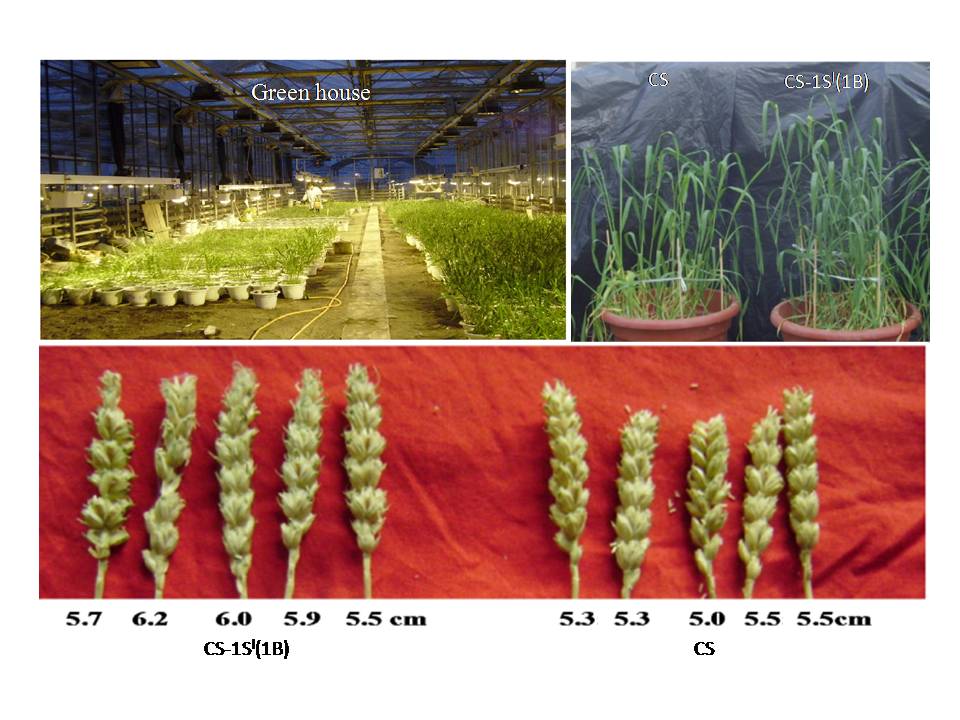

Supplement: Supplementary file 1 — Additional file 1: Figure S1. Performance of drought tolerance between CS and CS-1Sl (1B). [file 40529_2016_134_MOESM1_ESM.jpg]

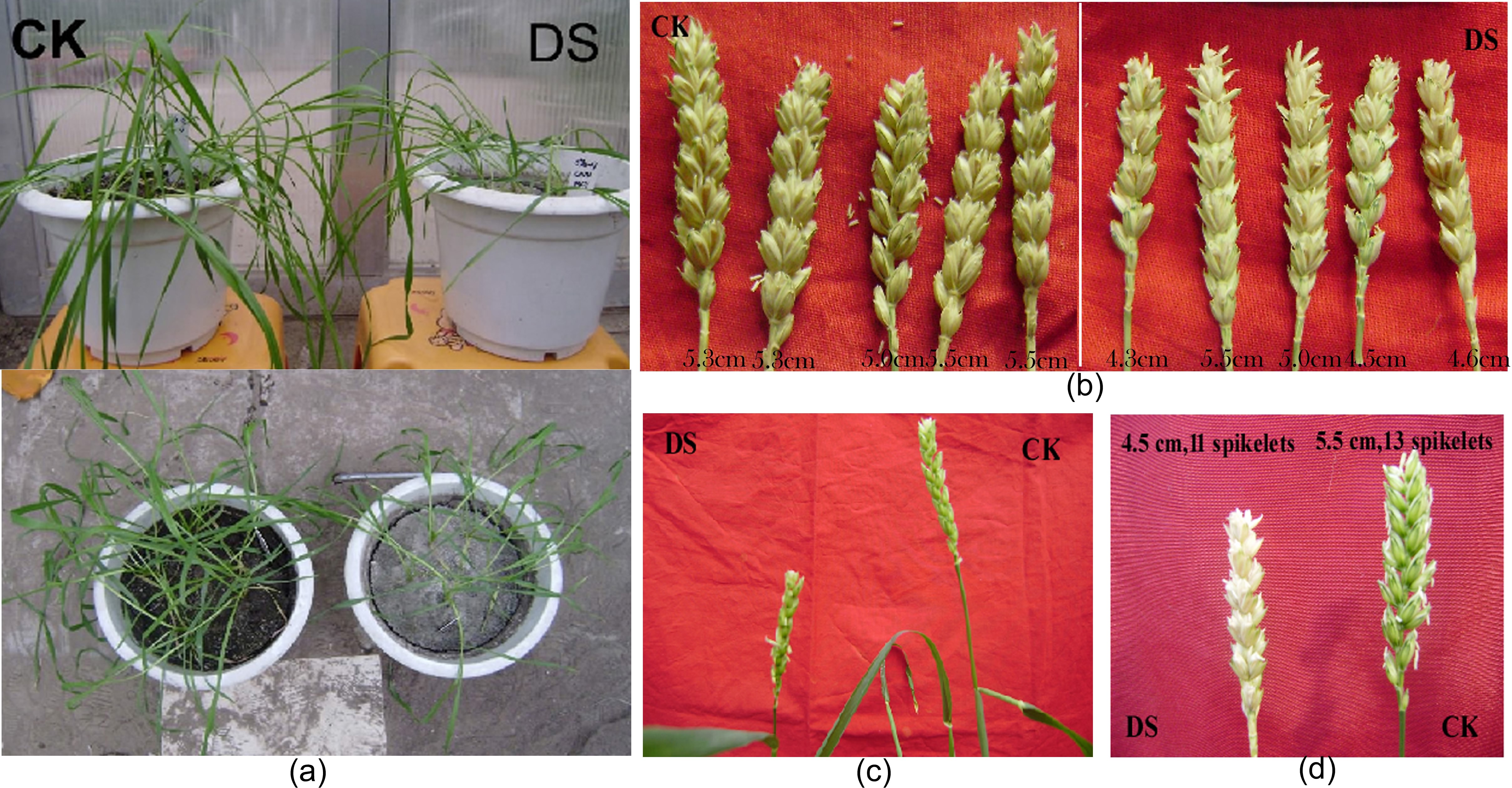

Supplement: Supplementary file 3 — Additional file 3: Figure S2. Pictures of CS-1Sl (1B) under drought stress and well-watered conditions in several grains development stages. (a).After tillering; (b). After harvest; (c). 5DPA; (d). 30 DPA. [file 40529_2016_134_MOESM3_ESM.jpg]

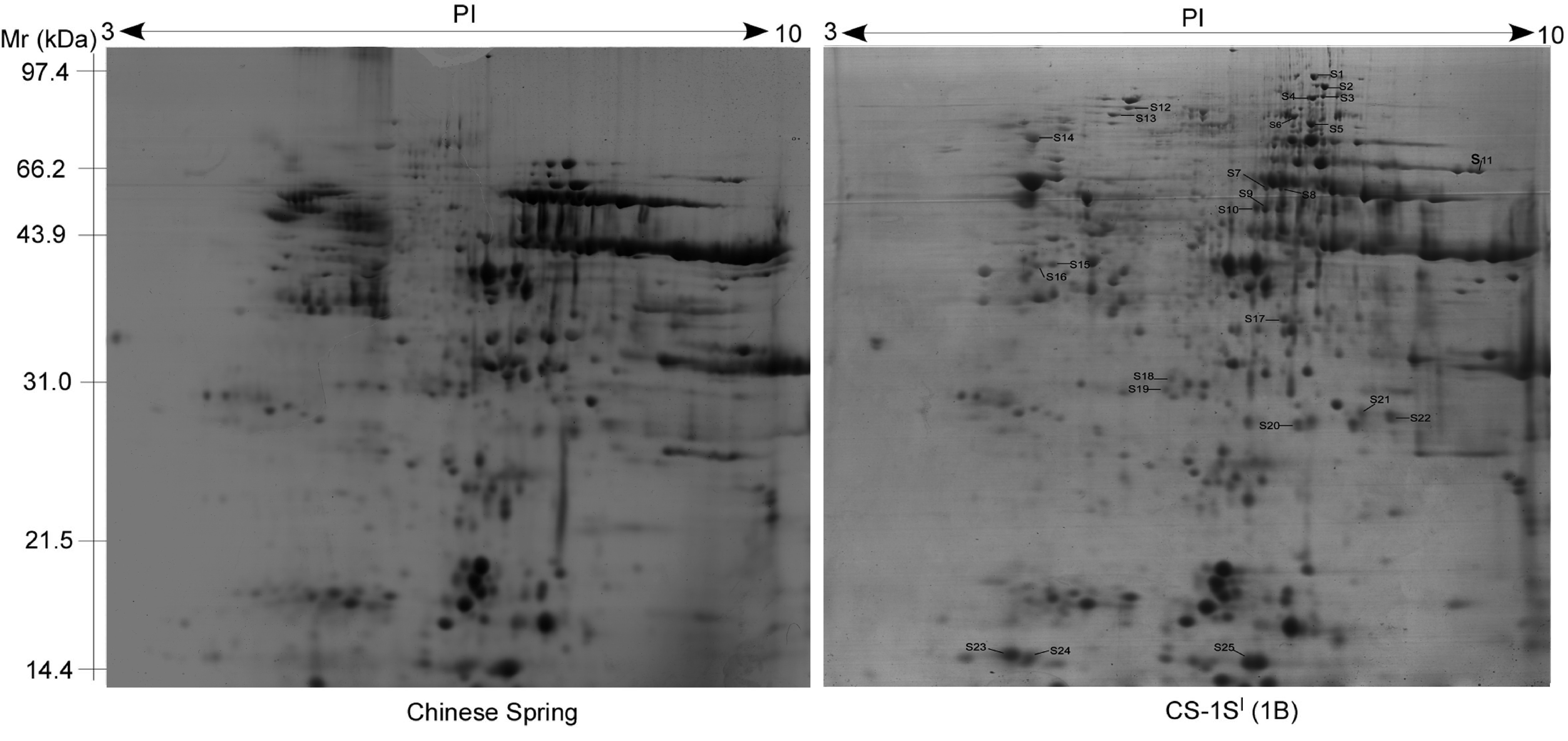

Supplement: Supplementary file 4 — Additional file 4: Figure S3. Proteome maps of wheat albumins and globulins from mature grains of CS and CS-1Sl (1B). S1 to S25 represented those specifically expressed in CS-1Sl (1B). The detail identification results were showed in Table1. [file 40529_2016_134_MOESM4_ESM.jpg]
