# Supplementary material for: Identification of drought stress related proteins from 1Sl(1B) chromosome substitution line of wheat variety Chinese Spring
Source: Bot Stud. 2016 Aug 9;57:20. doi: 10.1186/s40529-016-0134-x (PMC5430570; doi:10.1186/s40529-016-0134-x)
Supplement: Supplementary file 2 — Additional file 2: Table S1. Some agronomic character performance of CS-1Sl (1B) under drought stress and well-watered conditions. [file 40529_2016_134_MOESM2_ESM.doc]

**Table S1** Some agronomic character performance of CS-1S^l^ (1B) under drought stress and well-watered conditions

| Indices | CS-1S^l^(1B) | | |
| --- | --- | --- | --- |
|  | CK | | DS |
| Plant height(cm) | 67.6 | 62.6** | |
| Spike length(cm) | 5.32 | 4.8* | |
| Number of spikelet | 12.6 | 11.4* | |
| Grain number per spike | 17.4 | 12.7** | |
| Thousand kernel weight (g) | 24.67 | 22.67* | |
| Physiological development time (d) | 117 | 110 | |

*and ** indicate a significant difference at *P* < 0.05 and *P* < 0.01 level by t-test, respectively.
