# Supplementary material for: Identification of drought stress related proteins from 1Sl(1B) chromosome substitution line of wheat variety Chinese Spring
Source: Bot Stud. 2016 Aug 9;57:20. doi: 10.1186/s40529-016-0134-x (PMC5430570; doi:10.1186/s40529-016-0134-x)
Supplement: Supplementary file 5 — Additional file 5: Table S2. Peptide sequences of mature seed proteins encoded by 1Sl genome of CS-1Sl(1B) identified by MALDI-TOF/TOF-MS. [file 40529_2016_134_MOESM5_ESM.doc]

**Table S2** Peptide sequences of mature seed proteins encoded by 1S^l^ genome of CS-1S^l^(1B) identified by MALDI-TOF/TOF-MS

| Protein spot | Accession no.(gi) | Protein discription | ±da | ±ppm | Start Sequence | End Sequence | Peptide Sequence | Ion Score | Protein Score C.I.% |
| --- | --- | --- | --- | --- | --- | --- | --- | --- | --- |
| S2 | 39599016 | HMW glutenin subunit | -0.032 | -29 | 45 | 54 | QVVDQQLAGR | 91 | 100 |
|  |  |  | -0.0269 | -23 | 55 | 64 | LPWSTGLQMR | 83 | 99.998 |
|  |  |  | -0.0397 | -33 | 55 | 64 | LPWSTGLQMR | 40 | 62.105 |
|  |  |  | -0.0255 | -19 | 34 | 44 | ELQESSLEACR | 99 | 100 |
|  |  |  | -0.0257 | -17 | 635 | 647 | AQQPATQLPTVCR | 74 | 99.987 |
|  |  |  | -0.0042 | -2 | 136 | 157 | QGSYYPGQASPQQPGQGQQPGK | 102 | 100 |
|  |  |  | -0.0833 | -22 | 187 | 221 | QGYYPTSLQQPGQGQQIGQGQQGYYPTSPQHTGQR | 117 | 100 |
| S3 | 140169817 | Dy-type high molecular weight subunit protein | -0.0265 | -24 | 45 | 54 | QVVDQQLAGR | 78 | 99.994 |
|  |  |  | -0.0101 | -8 | 34 | 44 | ELQESSLEACR | 101 | 100 |
|  |  |  | -0.0158 | -11 | 629 | 641 | AQQPATQLPTVCR | 57 | 99.322 |
| S4 | 344995121 | y-type high molecular weight glutenin subunit | 0.0096 | 9 | 45 | 54 | QVVDQQLAGR | 71 | 99.967 |
|  |  |  | 0.0209 | 18 | 55 | 64 | LPWSTGLQMR | 56 | 98.973 |
|  |  |  | 0.0214 | 16 | 34 | 44 | ELQESSLEACR | 93 | 100 |
|  |  |  | 0.0285 | 19 | 726 | 738 | AQQPTTQLTTVCR | 86 | 99.999 |
| S5 | 344995121 | y-type high molecular weight glutenin subunit | -0.0419 | -38 | 45 | 54 | QVVDQQLAGR | 92 | 100 |
|  |  |  | -0.0157 | -12 | 34 | 44 | ELQESSLEACR | 102 | 100 |
|  |  |  | -0.0136 | -9 | 726 | 738 | AQQPTTQLTTVCR | 73 | 99.968 |
| S6 | 344995121 | y-type high molecular weight glutenin subunit | 0.0115 | 10 | 45 | 54 | QVVDQQLAGR | 85 | 99.999 |
|  |  |  | 0.0078 | 6 | 34 | 44 | ELQESSLEACR | 93 | 100 |
|  |  |  | -0.0107 | -7 | 726 | 738 | AQQPTTQLTTVCR | 83 | 99.998 |
|  |  |  | 0.0253 | 11 | 704 | 725 | QGYDSPYHVSAEQQAASSMVAK | 50 | 95.973 |
| S7 | 228310 | globulin 2 | -0.0408 | -38 | 96 | 105 | EGEGVIVLLR | 68 | 99.947 |
| S8 | 34495244 | globulin-like protein | -0.0328 | -30 | 122 | 131 | EGEGVIVLLR | 72 | 99.978 |
| S9 | 228310 | globulin 2 | -0.0358 | -33 | 96 | 105 | EGEGVIVLLR | 76 | 99.992 |
| S10 | 171027826 | triticin | -0.0471 | -42 | 202 | 210 | EFLFAGNYR | 56 | 99.23 |
|  |  |  | -0.0545 | -39 | 200 | 210 | HKEFLFAGNYR | 94 | 100 |
|  |  |  | -0.0553 | -34 | 132 | 146 | YGQSQSVQGQSQSQK | 65 | 99.901 |
|  |  |  | -0.0656 | -32 | 61 | 77 | SQAGLTEYFDEENEQFR | 151 | 100 |
|  |  |  | -0.0705 | -33 | 269 | 286 | EPESYPHTQYEEGQSQAK | 121 | 100 |
| S15 | 75279909 | Serpin-Z2B | -0.0898 | -97 | 11 | 18 | LSIAHQTR | 39 | 60.715 |
|  |  |  | -0.0768 | -64 | 182 | 191 | GAWTDQFDPR | 52 | 97.934 |
|  |  |  | -0.1088 | -86 | 289 | 300 | ISLGIEASDLLK | 41 | 76.11 |
|  |  |  | -0.0905 | -66 | 159 | 171 | DILPAGSIDNTTR | 70 | 99.966 |
|  |  |  | -0.0899 | -59 | 287 | 300 | FKISLGIEASDLLK | 41 | 75.889 |
|  |  |  | -0.0773 | -46 | 261 | 274 | LSAEPEFLEQHIPR | 108 | 100 |
|  |  |  | -0.0705 | -34 | 152 | 171 | VTTGLIKDILPAGSIDNTTR | 141 | 100 |
| S17 | 584706 | Aspartate aminotransferase | -0.0889 | -32 | 211 | 236 | ALLPFFDSAYQGFASGSLDQDAQSVR | 97 | 100 |
| S20 | 259122791 | APX | -0.0319 | -23 | 131 | 142 | LPDATKGCDHLR | 73 | 99.981 |
|  |  |  | -0.0737 | -47 | 2 | 14 | TKNYPAVSEEYQK | 47 | 93.12 |
|  |  |  | -0.0658 | -42 | 39 | 52 | LAWHSAGTFDCASR | 107 | 100 |
|  |  |  | -0.0609 | -38 | 210 | 223 | ALLDDPVFRPLVEK | 45 | 88.098 |
|  |  |  | -0.0605 | -31 | 62 | 79 | FDDELAHGANNGLHIALR | 87 | 100 |
|  |  |  | -0.0787 | -38 | 148 | 167 | QMGLTDQDIVALSGAHTL | 66 | 99.912 |
|  |  |  | -0.07 | -33 | 148 | 167 | QMGLTDQDIVALSGAHTLGR | 67 | 99.928 |
| S21 | 110341790 | globulin 1 | -0.0349 | -29 | 47 | 56 | QILEQQLTGR | 95 | 100 |
|  |  |  | -0.0346 | -23 | 99 | 111 | DYEQSMPPLGEGR | 55 | 99.128 |
|  |  |  | -0.0395 | -22 | 57 | 74 | AGEGAVGVPLFQAQWGAR | 158 | 100 |
|  |  |  | -0.0528 | -29 | 119 | 134 | QQEQGCSGESTEPEQR | 76 | 99.992 |
|  |  |  | -0.0451 | -23 | 182 | 200 | QQGEGFSGEGAQQKPQAGR | 154 | 100 |
|  |  |  | -0.0605 | -19 | 135 | 165 | QEVQGGQYGSETGGSQQQQQGGGYHGVTVGR | 224 | 100 |
| S22 | 110341790 | globulin 1 | -0.0258 | -22 | 47 | 56 | QILEQQLTGR | 100 | 100 |
|  |  |  | -0.0385 | -21 | 57 | 74 | AGEGAVGVPLFQAQWGAR | 146 | 100 |
|  |  |  | -0.0435 | -22 | 182 | 200 | QQGEGFSGEGAQQKPQAGR | 124 | 100 |
|  |  |  | -0.0319 | -10 | 135 | 165 | QEVQGGQYGSETGGSQQQQQGGGYHGVTVGR | 176 | 100 |
| S23 | 21711 | CM 17 protein precursor | -0.0089 | -8 | 46 | 54 | NYVEEQACR | 60 | 99.747 |
|  |  |  | -0.0075 | -4 | 92 | 107 | SRPDQSGLMELPGCPR | 49 | 96.575 |
|  |  |  | -0.0284 | -16 | 92 | 107 | SRPDQSGLMELPGCPR | 69 | 99.964 |
|  |  |  | -0.031 | -16 | 66 | 80 | QECCEQLANIPQQCR | 117 | 100 |
| S24 | 221855632 | alpha-amylase inhibitor CM16 | -0.0172 | -17 | 107 | 114 | EVQMDFVR | 63 | 99.83 |
|  |  |  | -0.0119 | -10 | 45 | 53 | DYVEQQACR | 76 | 99.992 |
|  |  |  | -0.0036 | -2 | 91 | 106 | SRPDQSGLMELPGCPR | 67 | 99.943 |
|  |  |  | -0.0223 | -12 | 91 | 106 | SRPDQSGLMELPGCPR | 59 | 99.614 |
|  |  |  | -0.0055 | -3 | 65 | 79 | QQCCGELANIPQQCR | 96 | 100 |
| S25 | 123957 | Alpha-amylase/trypsin inhibitor CM3 | 0.01 | 10 | 37 | 44 | TNLLPHCR | 48 | 93.983 |
|  |  |  | 0.0179 | 16 | 133 | 140 | EMQWDFVR | 70 | 99.963 |
|  |  |  | 0.0095 | 8 | 133 | 140 | EMQWDFVR | 49 | 95.414 |
|  |  |  | 0.0194 | 11 | 101 | 115 | YFIALPVPSQPVDPR | 86 | 100 |
|  |  |  | 0.0067 | 4 | 116 | 132 | SGNVGESGLIDLPGCPR | 134 | 100 |
|  |  |  | 0.0205 | 11 | 45 | 60 | DYVLQQTCGTFTPGSK | 79 | 99.996 |
|  |  |  | 0.013 | 7 | 141 | 157 | LLVAPGQCNLATIHNVR | 102 | 100 |
|  |  |  | 0.0115 | 6 | 81 | 95 | LYCCQELAEISQQCR | 113 | 100 |
